# A novel career development course for animal science students pursuing veterinary college admissions

**DOI:** 10.1093/tas/txab106

**Published:** 2021-07-01

**Authors:** Shweta Trivedi, Jessica C Clark, Dylan Deprospero, Kenneth Royal, Miles Todd See

**Affiliations:** 1 Department of Animal Science, North Carolina State University, Raleigh, NC 27695, USA; 2 Department of Clinical Sciences, College of Veterinary Medicine, North Carolina State University, Raleigh, NC 27695, USA

**Keywords:** career development course, pre-professional identity development, pre-veterinary advising, pre-veterinary curriculum, self-efficacy, vicarious experiences

## Abstract

A one-credit hour, elective, professional development course was created at North Carolina State University to introduce pre-veterinary track students to the admissions process and the breadth of the veterinary profession. The course was designed to facilitate career exploration while building self-efficacy through vicarious learning, interacting with speakers in various veterinary subfields, and addressing misperceptions about veterinary admissions. To evaluate the student learning objectives and improve upon the current practices of the course, data from two pretest and posttest course surveys for 235 course participants between Spring 2014 and 2017 were analyzed. The results of the study showed that students experienced significant gains in self-appraisal (Cohen’s *d* ranged 1.88 to 2.53), gathering occupational information (Cohen’s *d* ranged 1.59 to 2.53), goal selection (Cohen’s *d* ranged 2.14 to 2.53), and planning and problem-solving (Cohen’s *d* ranged 1.88 to 2.77) as well as experienced a decrease in five misperceptions about veterinary admissions. This novel course is presented as a prospective course for other universities.

## INTRODUCTION

To become a veterinarian, appropriate training and education must be obtained from a College of Veterinary Medicine (CVM), which has competitive admissions processes that vary per school (American Association of Veterinary Medical Colleges, 2020). The desire to meet the needs of the competitive admissions process contributes to a pattern where students focus solely on resume building rather than career exploration or professional development during their undergraduate career. Research shows that undergraduate students have a tendency to focus on perfectionism and building resumes rather than on career exploration; therefore, they exhibit a tendency to create a weak career identity (Sterle et al., 2016). These weak career identities cause concerns for students in their early graduate career when they are utilizing graduate school as their first opportunity for career exploration (Lehker and Furlong, 2006). Since the veterinary profession encompasses a vast array of fields and careers, students need to be aware of this variety and given the opportunity to explore different career options prior to pursuing a graduate degree.

Social cognitive theory states that students need to see themselves in a position before they can feel it is attainable. By seeing role models from similar backgrounds, individuals have increased feelings of self-efficacy (Bandura, 1977). Singer-Freeman and Bastone (2019) theorize that if we provide career exploration information during undergraduate education, then there are fewer barriers to diversifying identities within the science, technology, engineering, and mathematics (STEM) fields. Thus, the authors speculate that, by providing opportunities for students to engage with vocationally and racially diverse speakers, pre-veterinary students may be better able to develop their self-efficacy and to create attainable and personalized career goals.

### Course Description

At North Carolina State University (NCSU), a one-credit hour course is taught each spring for students considering a career in veterinary medicine. This course is titled ANS 281—Professional Development of Pre-Veterinary Track Students, and its overarching goal is to introduce students to the current veterinary admissions requirements and the scope of the veterinary profession. Pedagogically speaking, the course also seeks to provide students with opportunities to develop feelings of self-efficacy vicariously through invited guests who have successfully navigated veterinary education and are willing to share their candid experiences. Hands-on practice navigating the Veterinary Medical College Application Service (VMCAS), writing personal statements, and participating in mock interviews can provide individual experiences of mastery for students who have not had such experiences previously. Table 1 details the student learning objectives of the course. Indirectly, the incorporation of guest speakers into the curriculum offers students avenues for networking and seeking internship positions that will continue to provide efficacy-developing opportunities for the future. Finally, by reducing the “unknown” in the veterinary application process, the course aims to alleviate some of the student’s anxieties and resulting misperceptions about veterinary admissions.

**Table 1.** ANS 281 learning objectives related to self-efficacy aspects

Student learning objective	Self-efficacy aspect
Identify and critique current issues facing veterinary profession	Gathering occupational information
Create a career map for a successful admission into a veterinary school	Planning/Goal selection
Select internships to diversify animal and veterinary experiences for a competitive application	Planning/Goal selection
Create a VMCAS application	Gathering occupational information
Draft a personal statement and DVM interview questions for a successful application	Self-appraisal/Goal selection
Create a list of career options available to a veterinarian and analyze para-professional career paths in veterinary medicine	Gathering occupational information
Compare and contrast the role of a Dairy and Equine veterinarian to identify personal career goals	Gathering occupational information
Compare the diversity in the skill set of an Exotic animal veterinarian to identify personal career goals	Self-appraisal/Planning/Goal selection
Identify and assess the role of a Food Animal veterinarian and a Lab Animal veterinarian to select personal career goals	Gathering occupational information

The purpose of this research study was to: 1) describe a novel undergraduate professional development course to introduce students to veterinary admissions and career options within veterinary medicine and 2) evaluate the degree to which student learning objectives for the course were achieved.

## METHODS

### Participants and Procedures

Participants for this study involved four cohorts of students (2014–2017) enrolled in the aforementioned course. A total of 235 students were asked to complete two pretest and posttest questionnaires. The questionnaires were administered via paper-based survey to enrolled students in the classroom on the first and last days of the course. Students were told that completion of the questionnaires was completely voluntary, was not a graded assignment, and that all responses would remain anonymous. No incentives were offered in the form of extra bonus points or monetary gifts. Permission to conduct the study was granted by the university’s Institutional Review Board (IRB #14189).

Of the 235 students, 219 completed both questionnaires resulting in a 93.2% response rate. The class sizes for the four cohorts consisted of 46 (2014), 46 (2015), 67 (2016), and 60 (2017) students, respectively. With respect to demographic characteristics, 27 (12.3%) identified as male, 187 (85.4%) identified as female, 163 (76.5%) identified as White, 14 (6.6%) as Hispanic or Latino, 13 (6.1%) as Black or African American, 13 (6.1%) as Asian or Pacific Islander, and 10 (4.7%) as Other. The median age of participants was 20 yr old (M = 19.90, SD = 2.05).

### Instrumentation

In the field of career development, Taylor and Betz (1983) suggested measuring self-efficacy for five aspects of career decision-making behavior. These aspects included the ability to assess one’s strengths and weaknesses (*Self-appraisal*), the ability to learn about the different aspects of careers in which one has an interest (*Gathering occupational information*), the ability to choose a career path that is consistent with one’s interests and strengths (*Goal selection*), the ability to identify and implement a course of action toward one’s goals (*Planning*), and the ability to adjust when necessary (*Problem-solving*). Given this theoretical framework, a pretest and posttest questionnaire was developed in alignment with the student learning objectives that would both address the five aspects of career decision-making and assess the course content and goals as stated in the syllabus.

The instrument consisted of 10 categorical items measuring various aspects of self-efficacy, and five supplemental items measuring students’ misperceptions. The 10 categorical items (Table 2) utilized a 5-point rating scale with categories: 1 *= Not at all True*, 2 *= Not True*, *3 = Not Sure*, *4 = True*, and 5 *= Very True*. The Cronbach’s α reliability coefficient (Royal and Hecker, 2016) that measured internal consistency was 0.83 for both pretest and posttest scores. The five supplemental items used a variety of rating scale formats.

**Table 2.** List of self-efficacy pretest and posttest survey items

No.	Item	Self-efficacy aspect
Q1	I can describe various jobs that a veterinarian can do besides small animal practice.	Gathering occupational information
Q2	I can assess and critique current issues facing the veterinary profession.	Gathering occupational information
Q3	I can locate and select internships to diversify my animal and veterinary experiences.	Self-appraisal/Planning/Problem-solving
Q4	I can create a successful VMCAS application and personal statement when applying to Vet School.	Problem-solving
Q5	I can prepare myself well for a veterinary school interview when invited for one.	Self-appraisal/Problem-solving
Q6	I can identify areas for improving my experience portfolio to become a competitive applicant.	Self-appraisal/Problem-solving
Q7	I can explain to fellow PreVet track students what a large animal practitioner does.	Gathering occupational information
Q8	I can explain to fellow PreVet track students what a lab animal veterinarian does.	Gathering occupational information
Q9	I can identify and assess the suitability of the dual-degree programs in my future career path.	Self-appraisal/Goal selection
Q10	I can create a roadmap for myself for a successful admission into a veterinary program.	Planning/Goal selection

### Analysis

Data analyses for self-efficacy items consisted of calculating descriptive statistics and paired samples *t*-tests for pretest and posttest groups. Analysis of variance (ANOVA) techniques were used to compare performance relative to demographic variables. Significance testing was performed with alpha set to 0.05. Cohen’s *d* effect size estimates (Cohen, 1988) were calculated to determine the practical significance of any differences. It generally is inappropriate to compute an effect size based on the paired *t*-test value (Dunlop et al., 1996); therefore, the Cohen’s *d* effect size estimates computed for this analysis were based on the mean and SD scores from each group. Further, although the interpretation of Cohen’s *d* effect size estimates typically requires context-specific considerations, the generally accepted interpretation guidelines include 0.2 (small), 0.5 (medium), and 0.8 (large).

Data analyses for the supplemental misperceptions items were calculated using descriptive statistics. All data analyses were performed using SPSS statistical software (version 24.0).

## RESULTS

Student learning objectives for the course utilizing the pretest and posttest surveys served as the basis for evaluating the degree to which the course objectives were achieved. To discern achievement, posttest ratings would need to be higher than the ratings provided for pretest items.

Results indicate that statistically significant differences were discernible for all 10 categorical items of survey one (Table 3). When the mean difference was divided by the SD to calculate Cohen’s *d*, the authors found that course participants were able to improve their pretest and posttest scores by approximately 2 SDs. In all instances, students’ responses indicated gains in self-efficacy relating to self-appraisal, gathering occupational information, goal selection, planning, and problem-solving. In question 1, students exhibited an improvement in their self-efficacy in gathering occupational information with an average SD of 1.78. Question 2 showed, on average, an improvement in self-efficacy through gathering occupational information with about 1.98 SD. Question 3 exhibited an average increase in self-efficacy through self-appraisal, planning, and problem-solving by an average SD of 1.98. Self-efficacy through problem-solving indicated an increase in question 4, by an average SD of 2.77. Questions 5 and 6 showed increases in self-efficacy through self-appraisal and problem-solving by an average of 2.31 and 1.88 SDs, respectively. Questions 7 and 8 indicated an average increase of 1.59 and 2.53 SDs for gathering occupational information. Question 9 indicated that self-appraisal and goal selection were increased, on average, with an SD of 2.53. Lastly, question 10 indicated that students increased self-efficacy through planning and goal selection, on average, with an SD of 2.14. No statistically significant results were discernible based on students’ gender or race/ethnicity.

**Table 3.** A comparison of self-efficacy pretest and posttest survey results

No.	Pretest M (SD)	Posttest M (SD)	*t*	df	*P-*value	*d*
Q1	3.49 (.86)	4.79 (.48)	−23.185	217	0.000	1.78
Q2	2.57 (.84)	4.24 (.57)	−29.322	217	0.000	2.33
Q3	3.19 (.92)	4.66 (.51)	−21.856	216	0.000	1.98
Q4	2.18 (.88)	4.32 (.65)	−32.291	216	0.000	2.77
Q5	2.33 (.95)	4.26 (.70)	−29.452	216	0.000	2.31
Q6	3.19 (1.01)	4.72 (.55)	−22.108	217	0.000	1.88
Q7	3.25 (1.03)	4.57 (.57)	−20.393	217	0.000	1.59
Q8	2.47 (.93)	4.43 (.58)	−30.381	217	0.000	2.53
Q9	2.17 (.93)	4.22 (.67)	−28.892	217	0.000	2.53
Q10	2.91 (.88)	4.52 (.60)	−25.674	216	0.000	2.14

The results for the seven perception items exhibited some variability across pretest and posttest measurements (Table 4). The results indicate a 47.7% decrease in the number of participants who previously did not have a plan B or alternate career plan. There was a 63.2% decrease in students who did not know whether having a double major was favored by Doctor of Veterinary Medicine (DVM) admissions committees demonstrating that the misperception was successfully dispelled. The results showed a 62.5% decrease in students who did not know whether an advanced degree was favored by admissions committees, and a 97.1% decrease in students who did not know whether engaging in a study abroad was favored by admissions committees. Individual responses show that both of these misperceptions were eliminated. There was a 40.8% decrease in students who did not know whether multiple Graduate Record Examinations (GRE) attempts were favored by admission committees, and a 90.5% decrease in those who did now know whether the research was considered a critical component for DVM admissions. Participation in ANS 281 helped students dismiss both of these misperceptions successfully.

**Table 4.** A comparison of misperceptions pretest and posttest survey results

	Pretest	Posttest
How confident are you in your decision to become a veterinarian?		
Extremely	119	121
Reasonably	81	67
Somewhat	15	20
Not at all	3	9
Do you have a plan B (or an alternate plan)?		
Yes	153	183
No	65	34
In your opinion, does having a double major during undergraduate coursework get reviewed favorably by the DVM admissions committees?		
Yes	110	105
No	40	88
Don’t know	68	25
In your opinion, does having an advanced degree (Masters or a PhD) get reviewed favorably by the DVM admissions committees?		
Yes	155	175
No	7	22
Don’t know	56	21
In your opinion, does having a study abroad experience get reviewed favorably by the DVM admissions committees?		
Yes	181	193
No	8	19
Don’t know	28	6
In your opinion, do multiple GRE attempts (3 or more) get reviewed unfavorably by the DVM admissions committees?		
Yes	41	52
No	68	102
Don’t know	108	64
In your opinion, is gaining hands-on research experience one of the critical components of a successful DVM application?		
Yes	186	207
No	8	9
Don’t know	21	2

Since the questionnaires were de-identified, the authors used the course rosters from 2014 through 2017 to ascertain the total number of students who gained admission to CVM NCSU. Out of the DVM class of 2020–2024, 63 students from ANS 281 gained admission to CVM NCSU.

## DISCUSSION

According to Bandura’s (1977) Social Cognitive Theory, self-efficacy arises from four sources: 1) *personal experiences of mastery*, 2) *vicarious experiences of mastery*, 3) *social persuasion*, and 4) *physiological state.* By exposing students to diverse veterinary professionals and addressing any misperceptions related to the profession, the authors hypothesized that students would experience an increase in self-efficacy. Based on students’ pretest and posttest survey responses, the ANS 281 course appears to have improved student self-efficacy and corrected student misperceptions about veterinary admissions. Not only were all items statistically significant (*P* < 0.05), but all effect size estimates were also “large” in magnitude indicating substantial differences in scores. These findings are affirming of the course’s goals to raise student self-efficacy through gathering occupational information, improving goal planning and self-appraisal, and strengthening problem-solving and career planning skills.

All items in Table 4 exhibit a decrease in the number of students who “didn’t know” the answer to the five student misperceptions assessed. The importance of these data is to show that the majority of students gained a definitive answer for each of the misperceptions about career planning. Increasing discernment helps students with planning, goal selection, and problem-solving as they revise their own plans based on their improved understanding of the five misperceptions. The course was successful in dispelling the most commonly held misperceptions within the pre-veterinary student population.

The shift in the number of students who identified as confident or “not at all” confident in their decision to become a veterinarian is a successful indicator for this course. Encouraging students to explore a career prior to spending excess time and resources on training is important for both students who are planning to continue in a career path and those who realize their intended career does not align with their lifestyle or goals.

Perhaps the primary limitation of this study involves the self-reported nature of the survey. However, the pretest and posttest design of the study does increase the likelihood of success in measuring enhanced self-efficacy and decline in misperceptions. Another limitation involves the collection of anonymous data. Due to the anonymity of the data, the authors were unable to correlate success in the course to performance in other areas (i.e., the external aspect of validity).

Using Messick’s (1995) framework for interpreting construct validity, we found that theoretical expectations were supported by way of higher ratings on the posttest. This speaks to both the substantive and content aspects of construct validity. The response rate for students who completed both pretest and posttest questionnaires was 93.2%, which suggests that the likelihood for sampling bias was quite small. Also, the reliability coefficients were 0.82 to 0.83 for the pretest and posttest scores. These artifacts speak to the generalizability aspect of construct validity. Results indicated no statistically significant differences for students’ responses based on gender or race/ethnicity. The lack of differences supports the systematic aspect of construct validity. Finally, per admissions data provided by the Director of Student Services at CVM NCSU, nearly 40% of the in-state students enrolled in the DVM program each year were previously enrolled in this course. Considering that NCSU undergraduates comprise only 9% of the total reviewed applications for CVM NCSU, these data speak to the consequential aspect of construct validity. Collectively, there is valid evidence to support the author’s inferences and the general conclusion that the course has achieved its intended outcomes.

## CONCLUSIONS

The purpose of this study was to describe this preprofessional course and evaluate the course’s ability to instill self-efficacy and achieve its student learning objectives. The results based on four cohorts of students indicated that the course effectively increased students’ self-efficacy and decreased student misperceptions about DVM admissions. Students learned how to create personalized career maps (Supplementary Appendix 1), engaged with current veterinary professionals, corrected misperceptions about the veterinary admissions process, and practiced preparing materials for the VMCAS. Through these experiences, students had the opportunity to build self-efficacy and increase their VMCAS application’s competitiveness. The authors’ envision that the course described will serve as a model (Supplementary Appendix 2) for other institutions to incorporate career exploration, career planning, and vicarious learning experiences into their curriculum.

## Supplementary Material

txab106_suppl_Supplementary_Appendix
