# Supplementary material for: A novel career development course for animal science students pursuing veterinary college admissions
Source: Transl Anim Sci. 2021 Jul 1;5(3):txab106. doi: 10.1093/tas/txab106 (PMC8495951; doi:10.1093/tas/txab106)
Supplement: txab106_suppl_Supplementary_Appendix [file txab106_suppl_supplementary_appendix.docx]

Appendix 1: Student Sample of Career Map


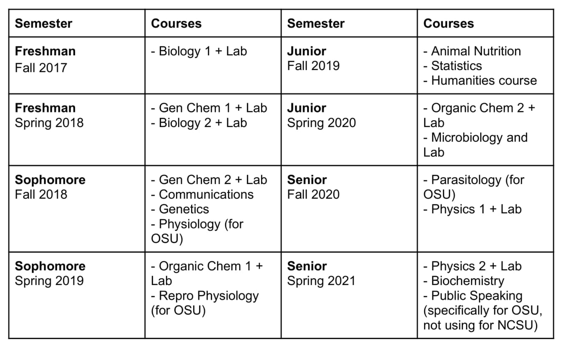


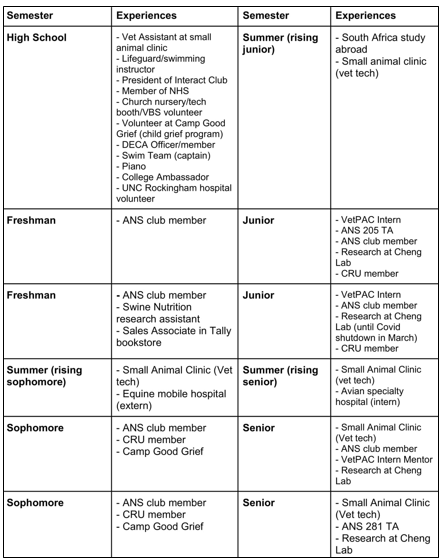


Appendix 2: Course Description

*Course description as model*

Since 2015, the average first year class of the Department of Animal Science at NCSU has consisted of 77% of students enrolled in the Veterinary BioScience concentration as pre-veterinary students. The ANS 281 course is a one credit hour, spring semester course with a current class size of 100 students to serve this population. While this elective course is primarily for second-year undergraduate students, it is open to all years and majors that are interested in pursuing a career in veterinary medicine. Course content focuses on career exploration, career mapping, VMCAS preparation, and networking. A faculty advisor teaches the majority of the curriculum related to preparing for the VMCAS, career planning, and career mapping. Community partners that serve as experts in their field provide the remainder of the career exploration curriculum. These guest speakers include institutional veterinarians such as the Chief Veterinarian from a local Museum to discuss exotic animal medicine, CVM faculty to introduce lab animal medicine, the CVM NCSU Director of Student Services teaches about DVM admissions, and a panel of current DVM students provides a student perspective on veterinary school. Additionally, the course hosts three teaching assistants (TA) whom are required to be current DVM applicants. Each TA is selected for their unique major, diverse career interests and background to provide multiple distinct role models for ANS 281 participants. Last, students are required to attend an annual pre-veterinary career networking event and the local CVM open house.

By the end of the course, students should be able to achieve the learning objectives outlined in Table 4.

These learning objectives assist with career exploration and encourage students to engage with a variety of careers that can assist with building self-efficacy.
